# Supplementary material for: Astronomical tuning of the Aptian stage and its implications for age recalibrations and paleoclimatic events
Source: Nat Commun. 2022 May 26;13:2941. doi: 10.1038/s41467-022-30075-3 (PMC9135687; doi:10.1038/s41467-022-30075-3)
Supplement: Supplementary file 1 — Supplementary Information [file 41467_2022_30075_MOESM1_ESM.pdf]

Supplementary Materials for:

Astronomical tuning of the Aptian Stage and its implications for age recalibrations and paleoclimatic events

C. G. Leandro*, J. F. Savian, M. V. L. Kochhann, D. R. Franco, R. Coccioni, F. Frontalini, S. Gardin, L. Jovane, M. Figueiredo, L. R. Tedeschi, L. Janikian, R. P. Almeida and R. I. F. Trindade

This supplementary document contains the following information:

Supplementary Figs. 1 to 5
COCO and eCOCO objective testing performed by means of Acycle software (version 2.4.1)

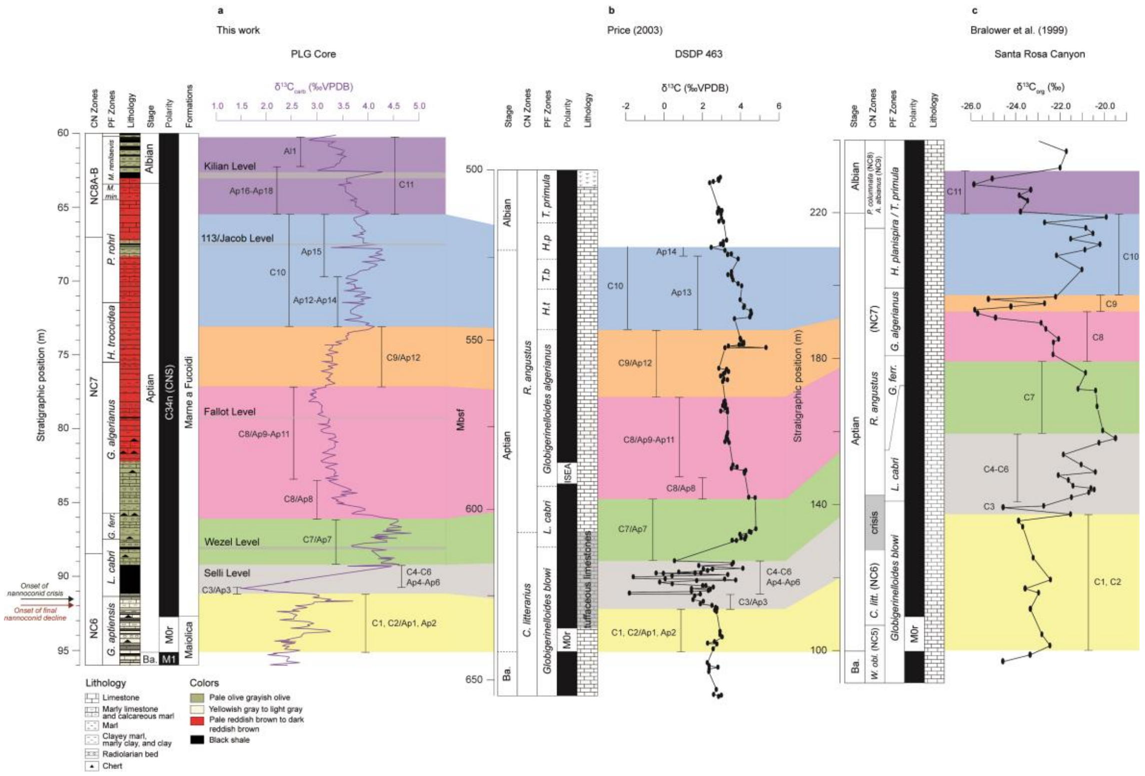

Supplementary Fig. 1 Stratigraphic framework and correlation between $\delta^{13}C$ data. **a** PLG core, depths for the upper boundaries of the planktonic foraminiferal, calcareous nannofossil zones, nannoconid decline and crisis biohorizons identified in the core[11,32] and this work. The gray bands highlight the Selli, Wezel, Fallot, 113/Jacob and Kilian Levels. Codes for C/Ap/Al-isotope segments[7,33]; **b** DSDP Site 463[35], codes for C/Ap-isotope segments[7,35]. The top of C10 segment is modified from Bottini et al.[7]**;** **c** Santa Rosa Canyon[34], codes for C-isotope segments[34]. The C/Ap segments are represented by same colors for three sites. Ba. Barremian, *M. Microhedbergella*, *min. miniglobularis*, *P. Paraticinella*, *H. Hedbergella*, *G. Globigerinelloides*, *ferr. ferreolensis*, *L. Leupoldina*, *R. Rhagodiscus*, *C. Chiastozygus*, *T. Ticinella*, *p. planispira*, *b. bejaouaensis*, *t. trocoidea*, *P. Prediscosphaera*, *A. Axopodorhabdus*, *litt. litterarius*, *W. Watznaueria*, *obl. Oblonga*.

COCO/eCOCO tests

Correlation coefficient (COCO) and evolutionary COCO (eCOCO) analyses[40] exhibited, for all tests (based on different sliding windows – 4, 6 and 8 m), two main maxima of stable sediment accumulation rate (SAR) at 0.52 and 0.58 cm/kyr with a correlation coefficient value exceeding 0.4 (Supplementary Fig. 2a, d, and g). SAR values are associated to null hypothesis significance levels (H_0, no orbital forcing) lower than 0.01 (Supplementary Fig. 2b, e and h), as well as to seven astronomical parameters involved in the estimation (Supplementary Fig 2c, f and i).

The eCOCO results (Supplementary Figs. 3 and 4) show two grouping solutions of potential sedimentation rate: (i) at 0.3–1 cm/kyr (peaks at 0.52 and 0.58 cm/kyr), and (ii) at 1.6–2.4cm/kyr (peak at 2.28 cm/kyr) (Supplementary Fig. 3a), all peaks with associated correlation coefficient values higher than 0.4. Although all SAR peaks exhibited H_0 significance levels lower than 0.01, it is noteworthy that the lowest results for the null hypothesis testing are evidenced by the 0.52 and 0.58 cm/kyr (lower than 0.001).

The evolution of contributing astronomical frequencies indicates that six terms are involved for the 1.6–2.4 cm/kyr sedimentation rates. In comparison, seven terms are involved at sedimentation rates of 0.3–1 cm/kyr (Supplementary Fig. 4c).

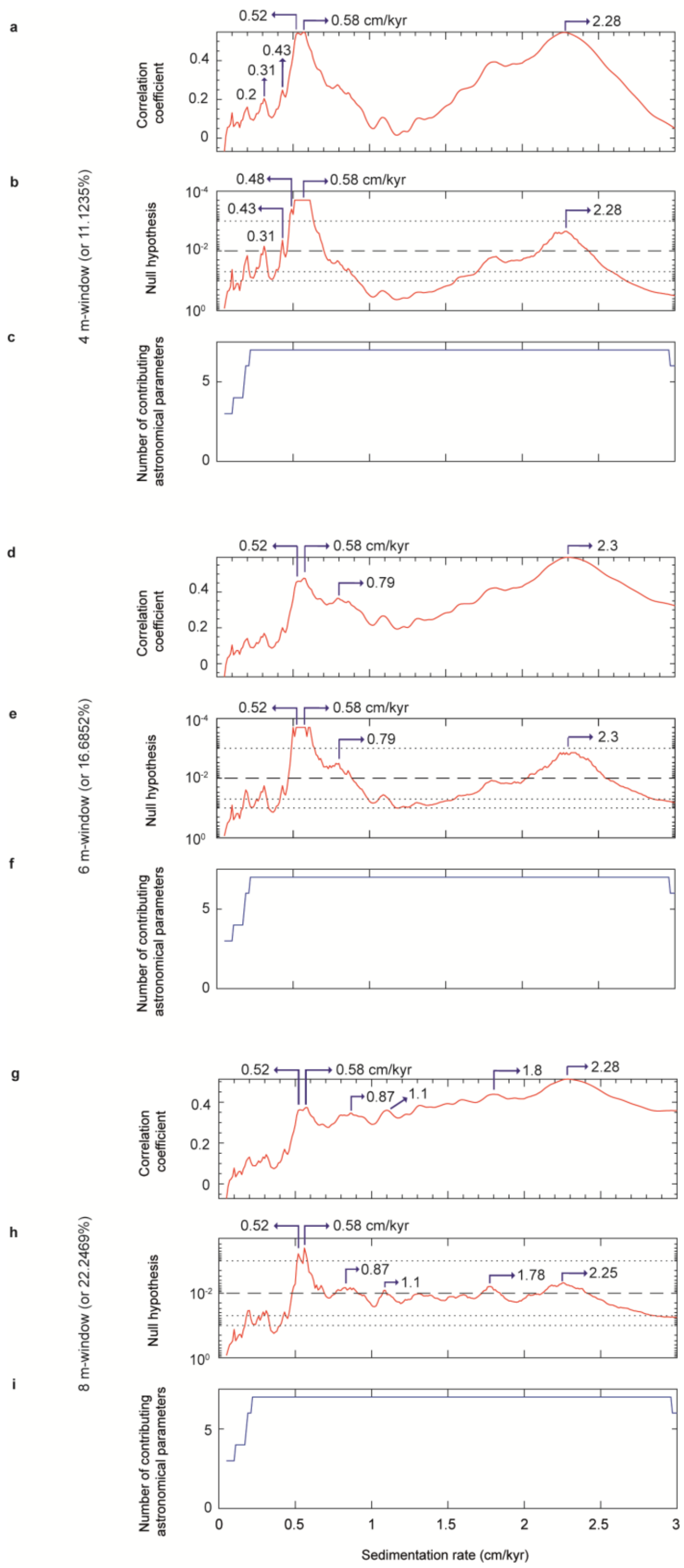

Supplementary Fig. 2. COCO analysis of the PLG core. **a**, **d**, **g** COCO analysis with labeled potential sedimentation rate for 4, 6 and 8 m windows. **b**, **e**, **h** Null hypothesis (H_0, no astronomical forcing) show 0.52 and 0.58 cm/kyr sedimentation rate, associated to H_0 significance levels lower than 0.01 for MS series. **c**, **f**, **i** are the number of contributing astronomical parameters in tested sedimentation rate. The target series is the La2004 astronomical solution[39] at 117 Ma. Significance levels are estimated by using Monte Carlo simulation of 5000 iterations with a step of 0.01 cm/kyr.

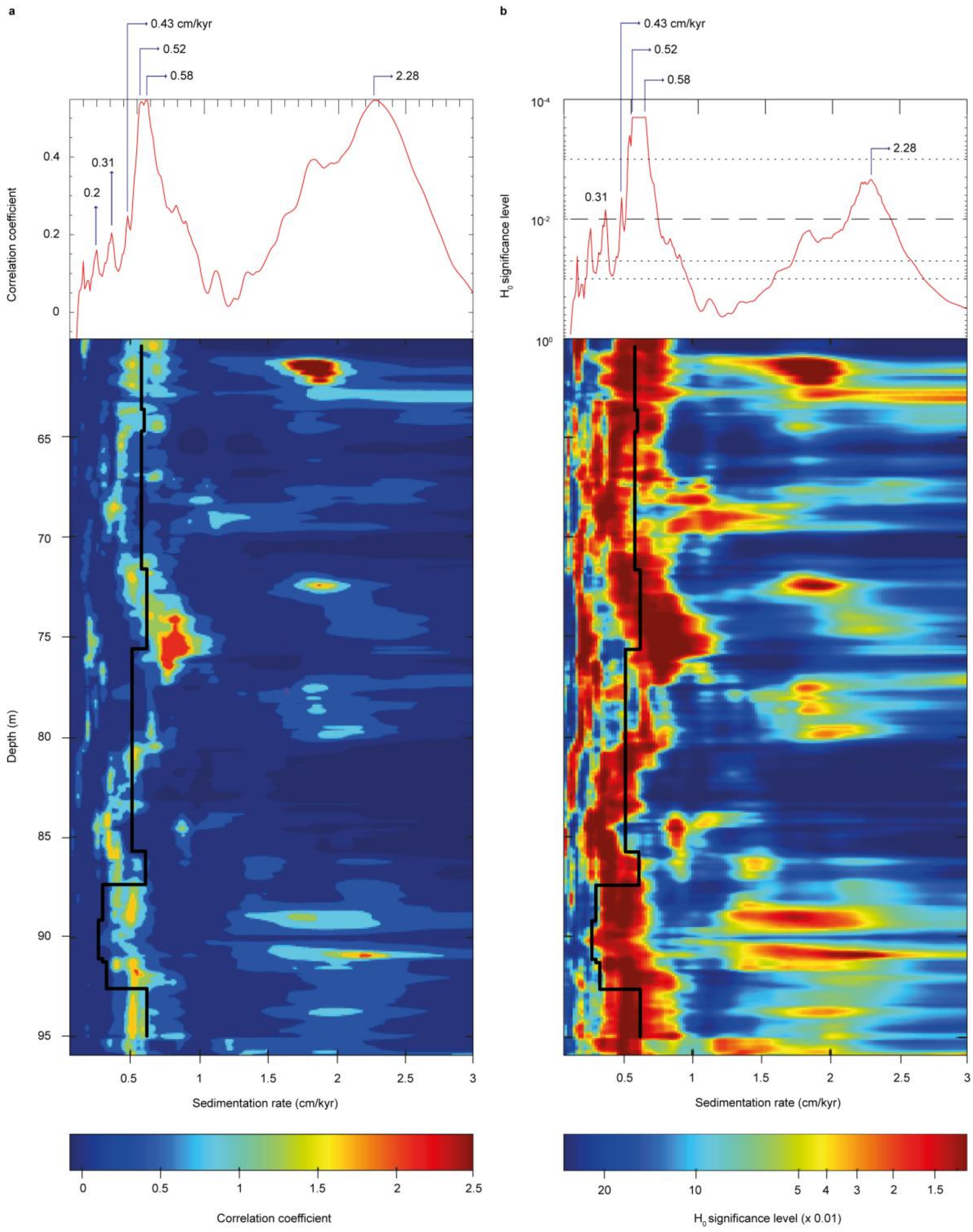

Supplementary Fig. 3. COCO analysis and eCOCO sedimentation rate map of the MS series in the PLG core. a Correlation coefficient (top) and evolutionary correlation coefficient (bottom, colored area) shown with sedimentation rate curve (black line) based on 405-kyr tuning. **b** Null hypothesis test (top) and evolutionary null hypothesis (H_0) significance level (bottom). For both the COCO and eCOCO analyses, tested sedimentation rates range from 0.05 to 3.00 cm/kyr and the number of Monte Carlo simulations is 5000. For COCO analysis the step is 0.01 cm/kyr and eCOCO analysis the sliding window size is 4 m with a step 0.12 m.

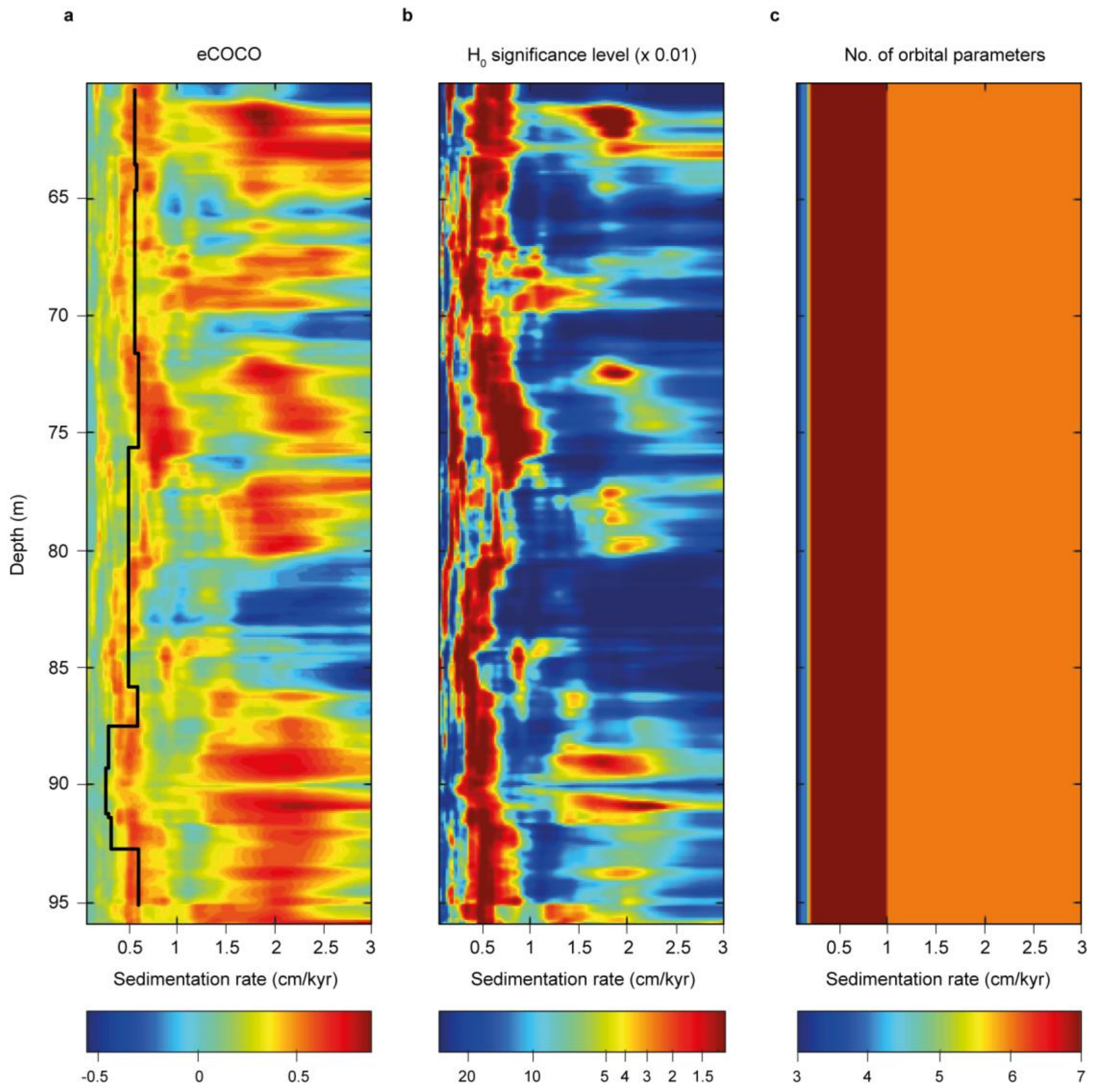

Supplementary Fig 4. eCOCO sedimentation rate map of the MS series in the PLG core. **a** Evolutionary correlation coefficient. **b** Evolutionary H_0 significance level. **c** Evolutionary map of the number of contributing astronomical parameters. The sliding window size is 4 m; the number of Monte Carlo simulations is 5000. Sedimentation rate from 0.05 to 3.00 cm/kyr with a step of 0.12 m. Our ATS (black line in a).

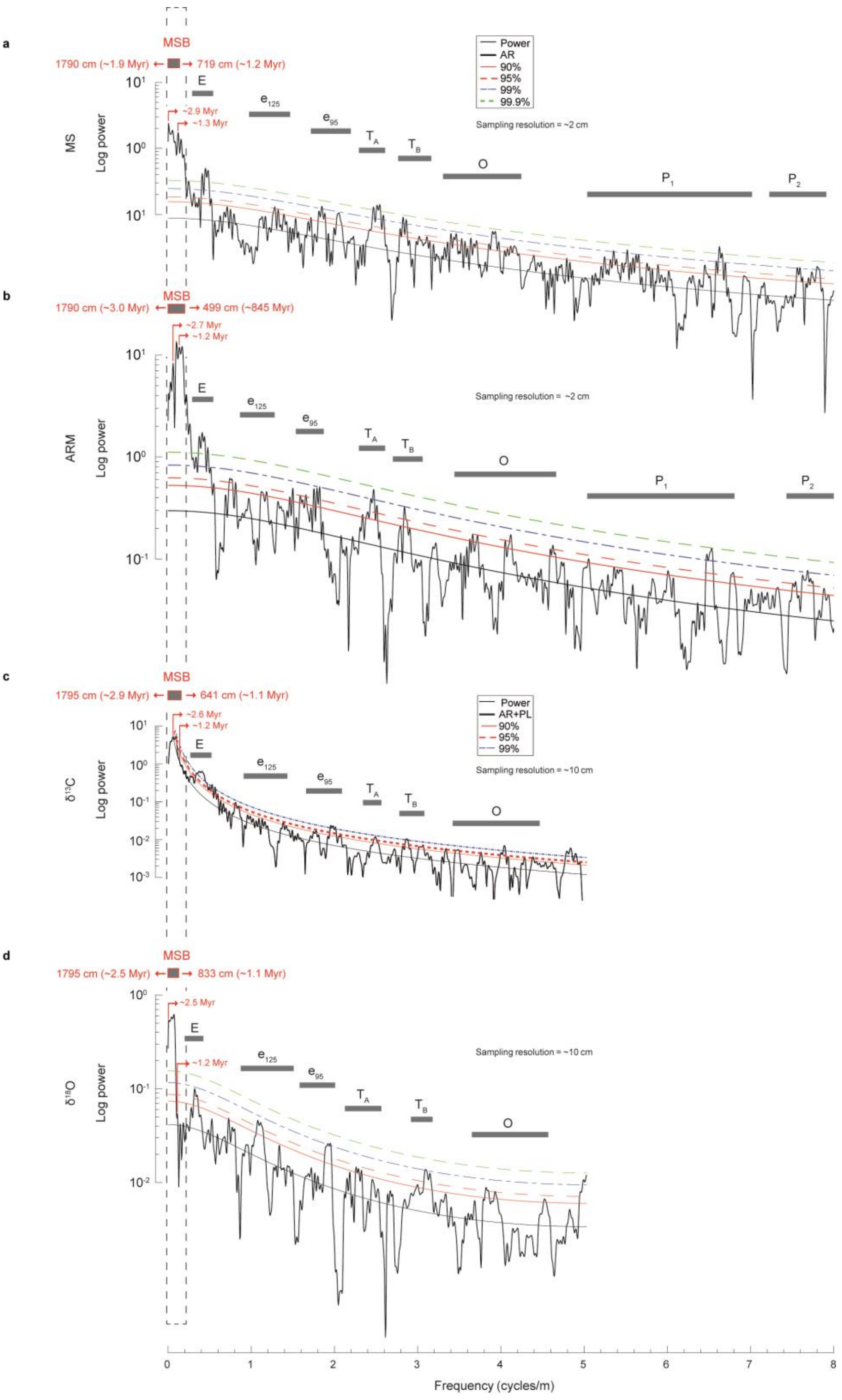

Supplementary Fig. 5 Spectral analysis of PLG core data. 2π multitaper power spectra **a** magnetic susceptibility (MS); **b** anhysteretic remanente magnetization (ARM); **c** $\delta^{13}C$; **d** $\delta^{18}O$ data, with the AR(1) red noise spectral model and 85%, 90%, 95%, and 99% confidence levels (c.l.) for null hypothesis testing. The rectangle (dashed line) indicates the frequency range for the MSB (million-year scale band). E long eccentricity, e_{125} and e_{95} 125-kyr and 95-kyr short eccentricity, T_A and T_B are referred to periodicities of ~60–70 kyr, O obliquity, P_1 and P_2 precession.
